# Integrating Orientation Optimization and Thermal Distortion Prediction in LPBF: A Validated Framework for Sustainable Additive Manufacturing

**DOI:** 10.3390/mi16111230

**Published:** 2025-10-29

**Authors:** Nikoletta Sargioti, Elias P. Koumoulos, Evangelia K. Karaxi

**Affiliations:** 1 Conify, P. Nikolaidi 23A, Agios Ioannis Rentis, 182 33 Athens, Greece; 2 IRES—Innovation in Research & Engineering Solutions, Silversquare Europe Square de Meeûs 35, 1000 Brussels, Belgium

**Keywords:** additive manufacturing, laser powder bed fusion, part orientation optimization, thermal distortion prediction, sustainability

## Abstract

This study investigates the impact of build orientation on thermal distortion, residual stress behaviour, and process efficiency in LPBF. Four orientation strategies, optimized for surface area, support volume, print time, and overheating, were generated in Siemens NX and evaluated using Atlas 3D to predict build-stage and post-support removal distortion. Experimental validation through 3D scanning enabled detailed surface deviation comparisons with simulation outputs. Results showed that support volume and print time optimizations led to the lowest in-process distortion but exhibited higher deformation after support removal, driven by residual stress relaxation. In contrast, the surface area-optimized orientation displayed greater distortion during printing but more stable post-processing behaviour. The overheating-optimized build resulted in the largest total distortion. Atlas 3D predictions aligned closely with scan data, particularly in identifying critical zones on sloped and unsupported surfaces. Sustainability and cost analysis revealed that the surface area strategy had the highest impact in reducing CO_2_ emissions and production costs (~€832 and ~900 g CO_2_/part), while support volume and print time orientations reduced cost by more than 20% and halved emissions. Energy consumption followed the same trend, with support volume and print time optimisations requiring only ~2 kWh/part compared to nearly 5 kWh/part for surface area, and overheating minimisation. These findings underscore the importance of integrating distortion simulation, cost, and environmental criteria into orientation selection to achieve balanced, high-performance LPBF manufacturing.

## 1. Introduction

Additive Manufacturing (AM), particularly Laser Powder Bed Fusion (LPBF), has improved the production of complex and lightweight metal components. By building parts layer by layer, LPBF enables intricate geometries, reduced material waste, and enhanced customization potential. However, the quality and manufacturability of LPBF parts are significantly influenced by part orientation, which affects printability, mechanical performance, and post-processing requirements. Part orientation in LPBF is a critical factor that impacts residual stress distribution, microstructure, and mechanical properties of the fabricated components. Studies have demonstrated that different build orientations can lead to variations in residual stress magnitudes and distributions. For instance, vertically built samples often exhibit higher tensile residual stresses, while horizontally built samples may present higher compressive residual stresses [1]. These residual stresses can adversely affect fatigue properties and dimensional accuracy, necessitating additional manufacturing operations such as stress-relief heat treatments [2]. Achieving first-time-right (FTR) manufacturing in Laser Powder Bed Fusion (LPBF) is strongly influenced by the interplay between build orientation, support design, and distortion control [3]. Residual stresses from steep thermal gradients often cause warping and dimensional deviations, particularly after support removal or post-processing. Orientation is a primary lever for distortion mitigation because it dictates heat flow, overhang stability, and residual stress distribution. However, orientation decisions involve inherent trade-offs: minimizing support volume and build time typically increases distortion risk, while orientations that reduce distortion often require more supports, longer build times, and higher costs. Recent studies advocate simulation-driven orientation selection, where thermo-mechanical models predict both in-process and post-release deformation, enabling pre-deformation compensation and reducing trial-and-error iterations [3,4].

Support structures add another layer of complexity to this trade-off triangle. While stiff supports improve in-process stability, they can amplify spring back after removal; conversely, compliant supports ease removal but allow greater distortion during printing [5]. Hybrid support strategies—stiff in critical zones, compliant elsewhere—are emerging as best practice to balance these competing objectives. Multi-objective optimization frameworks now integrate orientation, support topology, and sustainability metrics, demonstrating significant reductions in build time and material usage without compromising accuracy. Despite these advances, gaps remain in fully coupling distortion prediction with design-for-additive-manufacturing (DfAM) guidelines and accounting for post-processing effects [3]. Ultimately, achieving FTR in LPBF requires early-stage design decisions informed by predictive modelling and explicit consideration of the orientation–support–distortion trade-off triangle, alongside cost and environmental impact [4].

Moreover, build orientation influences the microstructural evolution during the LPBF process [6]. The direction of layer deposition affects grain morphology and texture, which in turn impact mechanical properties like tensile strength, hardness, and elongation at break. Certain orientations may promote the formation of columnar grains aligned with the build direction, leading to anisotropic mechanical behavior [7]. These grain structures, along with internal porosity variations, affect the fatigue life and long-term durability of the components, particularly in aerospace and biomedical applications where structural reliability is crucial. Therefore, selecting an optimal build orientation is essential for achieving desired mechanical performance and minimising residual stresses in LPBF-fabricated parts [8].

Recent advancements in topology optimization and part orientation strategies have aimed to enhance the quality and efficiency of LPBF processes. Miki and Yamada proposed a topology optimization method that considers distortion in additive manufacturing. Their approach involves a computationally inexpensive analytical model that accounts for residual stress and distortion during the LPBF process, facilitating the design of geometries less susceptible to manufacturing-induced deformations [9]. Such approaches reduce the need for post-processing and increase the efficiency of the manufacturing cycle by integrating structural considerations early in the design phase. Additionally, machine learning-based models have been explored for orientation optimization, leveraging data-driven approaches to predict and mitigate distortions before fabrication. In the context of AM, the integration of build orientation design with topology optimization is important for enhancing structural performance and manufacturability. The study by Zhou et al. presents a multicomponent topology optimization method that simultaneously optimises structural topology, partitioning, and build orientations of components. This approach accounts for anisotropic material behavior due to build orientation and imposes stress constraints at component interfaces, leading to assemblies with improved stiffness and reduced likelihood of failure at joints [10]. By considering anisotropic properties and interface stresses during the design phase, this methodology facilitates the production of components with superior mechanical performance and reliability, particularly in applications where structural integrity is critical. Malbašić et al. proposed a framework for additive manufacturing process planning that simultaneously optimises topology and part orientation. Their study demonstrated that simultaneous optimization reduces production time and cost by balancing computational software-driven solutions with operator experience in selecting optimal part orientation. By using multi-criteria decision-making (MCDM) methods, their framework enables improved part orientation through factors such as total build time, build cost, support volume, and support surface [11]. The study highlighted the importance of alternative build orientations (ABOs) and optimal build orientations (OBOs) in reducing production inefficiencies and improving manufacturability.

Additionally, studies have investigated the effect of build orientation on the mechanical and electrical properties of materials fabricated by Electron Beam Powder Bed Fusion (E-PBF). Research on pure copper components produced at different orientations found that build orientation significantly influences the microstructure, which in turn affects mechanical and electrical properties [12]. These findings underscore the importance of strategic orientation selection to optimise the functional performance of additively manufactured parts. Furthermore, studies have shown that by integrating finite element modelling (FEM) with experimental validation, predictive models can be developed to pre-emptively address issues related to thermal gradients and distortion in AM-produced parts, thus improving overall reliability and efficiency [13].

Several studies have explored part orientation optimization and its effects on LPBF-printed components, particularly in relation to residual stress, thermal distortion, and mechanical performance. Wang et al. proposed a multi-objective optimization framework that considers both thermal distortion and support structure minimization in AM processes [14]. While their method effectively reduced material waste and improved part stability, it primarily relied on computational modeling, leaving a gap in experimental validation. Similarly, Dinesh and Sahu investigated distortion and residual stress in LPBF-printed Inconel 718 parts using thermomechanical simulations in SIMUFACT [15]. Their findings emphasized the role of heat treatment in reducing residual stresses but did not explore how optimized part orientation could mitigate these distortions before fabrication.

Beyond computational studies, experimental validation has been recognized as an essential step in improving predictive models. Minetola et al. conducted one such validation study, comparing numerical simulations with actual printed components, demonstrating that unvalidated models may lead to deviations from real-world distortion behavior [16]. This highlights a broader issue in the literature: many optimization studies focus on theoretical solutions without empirical testing, limiting their practical applicability. Morgan et al. investigated part orientation strategies primarily to reduce support structures in LPBF, but their study did not account for thermal distortions, an equally critical factor influencing manufacturability [17]. A broader review by Qin et al. reinforced this limitation, noting that many computer-aided part orientation methods fail to integrate manufacturability constraints with thermal distortion predictions, often treating them as separate optimization problems [18].

Despite significant advancements in part orientation optimization and thermal distortion prediction, existing research often treats these aspects independently, lacking a unified framework that integrates orientation selection with distortion analysis. Moreover, many studies rely heavily on simulations without experimental validation, leaving uncertainty regarding the accuracy of predictive tools in real-world applications. Additionally, the impact of part orientation on residual stresses and distortions in complex, topology-optimized structures remains underexplored. Traditional trial-and-error methods for orientation selection further hinder efficiency in LPBF manufacturing.

Siemens NX is a comprehensive software platform widely used in additive manufacturing for optimising part orientation and minimising support structures, contributing to improved manufacturability and reduced distortion. Atlas 3D’s Sunata™ software further enhances this process by providing automated orientation selection based on thermal distortion analysis, aiding in reducing residual stresses and improving part accuracy. Unlike conventional thermal-mechanical simulations that rely on extensive Finite Element Modelling (FEM), Atlas 3D uses a Thermal Circuit Network (TCN) model to rapidly predict distortions in LPBF processes. This method is based on the lumped parameter modelling approach, where the part is divided into multiple thermal circuit elements (TCEs) instead of a fine computational mesh. Each TCE represents a uniform thermal volume, connected through thermal resistances, which approximate heat flow and temperature gradients. The TCN model enables fast, computationally efficient distortion predictions, making it suitable for real-time manufacturability assessments [7]. Once the thermal history of the part is generated, it is assigned to an FEM mesh, allowing a quasi-static thermo-mechanical model to estimate distortions both during and after printing. This hybrid approach enables Atlas 3D to provide fast and accurate predictions of thermal distortions, which can be used to refine build orientations for improved manufacturability [7]. According to Peng et al., the TCN model predicts the thermal history of metal PBF AM parts more than 100 times faster than conventional FEM simulations, with an accuracy reduction of less than 15% [19].

In addition to mechanical performance and thermal stability, sustainability and cost efficiency are critical considerations in LPBF manufacturing. The energy-intensive nature of LPBF processes, along with the consumption of expensive metal powders and post-processing resources, underscores the need for optimising build strategies not only for quality but also for environmental and economic impacts. Build orientation plays a crucial role in influencing material usage, support volume, energy consumption, and overall manufacturing costs. Orientations that minimise support structures and build times can substantially reduce embodied energy, CO_2_ emissions, and operational expenses [20,21]. Studies have shown that integrating sustainability considerations into AM process planning leads to significant reductions in resource usage without compromising part performance [22]. Furthermore, recent frameworks for LPBF production planning emphasise the importance of simultaneously optimising topology and part orientation to achieve substantial reductions in both build time and production costs [11]. These integrated approaches, which incorporate design for manufacturability, feature recognition, and MCDM methods, demonstrate that sustainable and cost-effective AM production is best achieved through holistic planning early in the design phase. Therefore, a comprehensive evaluation of part orientation must consider not only distortion mitigation and mechanical integrity but also the associated sustainability and cost implications, supporting more responsible and efficient manufacturing practices.

This study addresses these gaps by employing orientation optimization (Siemens NX) with thermal distortion prediction (Atlas 3D) on a topology-optimised motor bracket. We examined four orientation strategies (minimised supported surface area, minimised support volume, minimised print time, and overheating control). Simulations were performed for all four orientations; a single build (the overheating-optimised orientation) was manufactured by LPBF to represent worst-case validation. The printed part was stress-relief heat-treated and post-processed (support removal), and 3D scans were acquired both on-plate and after support removal. These data were compared against Atlas 3D predictions; in parallel, we quantified sustainability and cost implications for each orientation. Altogether, the workflow demonstrates how data-driven, simulation-assisted planning can enable “right-first-time” manufacturing, minimise trial-and-error iterations and post-processing, and improve environmental and economic performance.

Beyond the specific tools used, this work contributes a validated, transferable workflow for orientation decision-making in LPBF that integrates (i) multi-objective orientation screening, (ii) rapid distortion prediction, (iii) experimental verification using deviation analysis at two process stages, and (iv) sustainability/cost assessment. It is demonstrated that orientation optimization in LPBF is inherently a multi-objective problem that extends beyond geometric considerations. Effective decision-making requires a single decision-making framework, addressing the current fragmentation in LPBF research, that integrates build-stage and post-support removal distortion behavior, residual stress relaxation, and sustainability metrics such as cost, energy, and CO_2_ emissions. Our findings highlight that minimizing in-process distortion alone can lead to suboptimal outcomes after support removal, and that trade-offs between dimensional accuracy and environmental impact are unavoidable. Furthermore, the analysis highlights critical trade-offs between geometric accuracy and sustainability, demonstrating that strategies optimized for distortion control often conflict with those aimed at reducing cost and environmental impact. Finally, by experimentally validating simulation predictions through 3D scanning, we establish a reliable, simulation-driven workflow that reduces reliance on trial-and-error approaches and enables more efficient and sustainable LPBF process planning.

## 2. Materials and Methods

### 2.1. Case Study—Motor Bracket

**Figure 1:**
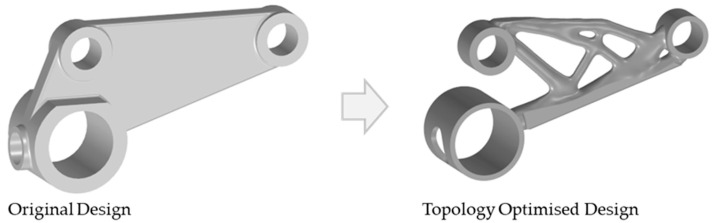
Comparison between the original and topology optimised design of the motor bracket. The topology optimised design (right) features a lightweight structure with reduced material usage, achieving a ~62% weight reduction, while maintaining the mounting interfaces (load-bearing features) for structural integrity, making it suitable for additive manufacturing. This optimised geometry is used in the study for orientation selection and thermal distortion analysis.

To evaluate the effectiveness of part orientation optimization and thermal distortion prediction in additive manufacturing, a motor bracket was selected as the case study. This component is commonly used in automotive and aerospace applications, where lightweight structures with high strength are required. The bracket features a topology optimised design, characterised by thin sections, making it particularly prone to residual stresses and distortions during the LPBF process. The geometry of the motor bracket consists of cylindrical mounting features and structural arms, which introduce overhangs and unsupported regions when oriented for printing. These characteristics make it an ideal candidate for orientation optimization, as different orientations will significantly impact support volume, print time, and thermal accumulation. For this study, AlSi10Mg was selected as the build material due to its widespread use in LPBF for lightweight structural applications, offering a good balance of mechanical properties, thermal conductivity, and printability. The goal of this study is to determine the optimal build orientation using Siemens NX, followed by verification of thermal distortions using Atlas 3D. An image of the motor bracket is provided in Figure 1, illustrating its complex design and structural features. This case study allows for a realistic evaluation of the effectiveness of computational optimization tools in reducing support material, minimising thermal distortions, and improving overall manufacturability.

### 2.2. Simulatio-Based Orientation Selection

In this study, Siemens NX (version 2412) was employed to determine the optimal build orientation for additive manufacturing components. Within the Modeling application of Siemens NX, the ‘Optimize Part Orientation’ tool—accessible under the ‘Design for Additive Manufacturing’ suite—was utilised to enhance print quality and efficiency.

The ‘Optimize Part Orientation’ tool systematically evaluates multiple orientations of a part to identify the most favorable configuration based on specific criteria. Users can assign weighting factors to each criterion, allowing prioritization based on the specific requirements of the manufacturing process. These factors include:

- Minimised Support Volume: Reducing the amount of support material necessary, thereby decreasing material consumption and simplifying post-processing efforts.
- Reduced Build Time: Lowering the overall printing duration by optimising the part’s positioning to achieve efficient layering and scanning sequences.
- Overheating Control: Mitigating thermal accumulation and potential overheating by selecting orientations that promote uniform heat distribution during the printing process.
- Minimised Surface Area Needing Support: Reducing the extent of part surfaces that require additional support, further minimising material waste and post-processing time.

Each of these criteria can be assigned different weighting factors, allowing the optimization process to focus on the most critical parameters based on the specific requirements of the build. By adjusting these factors, users can influence the ranking of possible orientations and select the best trade-off between manufacturability and performance. The tool provides results in terms of key metrics for each evaluated orientation, presenting a range of values, including minimum, actual, and maximum for each selected criterion. This enables an informed selection process, balancing different optimization goals such as reduced printing time, lower support volume, and improved thermal stability.

In this study, four separate part orientation optimizations were conducted, each prioritising one of the four parameters: surface area, support volume, print time, and overheating. For each optimization run, the weighting factor for the selected parameter was set to 100, while the remaining three parameters were assigned a weighting factor of 0. This ensured that each optimization exclusively focused on a single parameter at a time. Siemens NX provided a set of 10 possible orientations for each optimization run, ranked based on the chosen criterion. The first-ranked orientation from each optimization study was selected for further evaluation in the verification and validation stages of the workflow.

**Figure 2:**
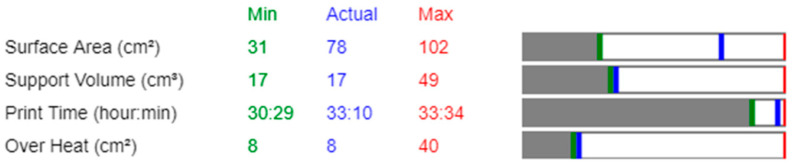
Example of multi-objective visualization of Siemens NX part orientation optimization results. The chart presents trade-offs between surface area, support volume, print time, and overheating, allowing for an informed selection of the most suitable build orientation.

The results from the Siemens NX part orientation optimization are presented in a multi-objective format, displaying the trade-offs between different criteria. The software provides a visual representation of the optimization results, indicating the minimum, maximum, and actual values for each evaluated parameter—surface area, support volume, print time, and overheating. A bar chart illustrates the range of possible values, where the green line denotes the minimum value, the red line represents the maximum value, and the blue line marks the actual value for the currently selected orientation. The grey area signifies non-feasible values, while the white area encompasses the feasible optimization range. These visual indicators enable users to compare different orientations objectively and select the most suitable configuration based on the specific manufacturing constraints (Figure 2). In this study, the selected orientations were analysed to ensure that the actual values were as close as possible to the minimum for each optimization parameter, thereby reducing material waste, minimising build time, and mitigating overheating risks. The results from this optimization were later compared with Atlas 3D outputs to validate the effectiveness of the orientation selection in minimising thermal distortions and improving overall printability.

In this study, the selected orientations were analysed to ensure that the actual values were as close as possible to the minimum values for each optimization parameter, thereby reducing material waste, minimising build time, and mitigating overheating risks. The results from Siemens NX’s part optimization were compared to Atlas 3D outputs to assess the thermal distortions associated with the selected orientations. The optimal part orientations determined in Siemens NX were exported and imported into Atlas 3D to generate distortion predictions and evaluate their thermal deformation behaviour. Atlas 3D employs a TCN model to predict heat accumulation and distortion trends. Using this approach, a distortion risk map was generated for each imported orientation, providing insight into potential deformation areas. This comparison allowed for an evaluation of how the optimized build orientations influenced thermal distortions, ensuring that manufacturability assessments accounted for both print efficiency and distortion behaviour.

### 2.3. Additive Manufacturing Build Preparation

**Figure 3:**
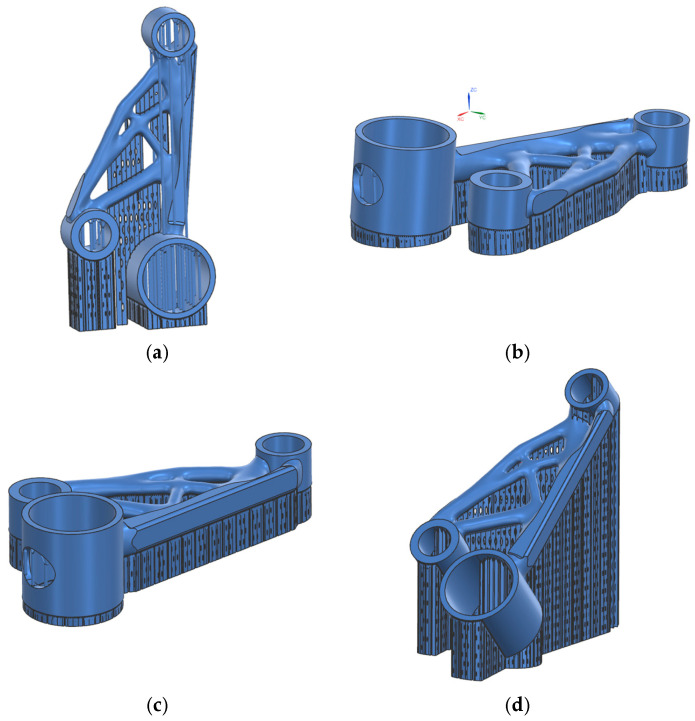
Support structures applied to different part orientations optimised using Siemens NX. (**a**) Surface area optimisation with tree supports and perforated block & line supports. (**b**) Support volume optimisation with tree supports and perforated block supports. (**c**) Print time optimisation with tree supports and perforated block supports. (**d**) Overheating optimisation with point supports and perforated block & line supports.

The build preparation process was conducted using the Siemens NX Additive Manufacturing tool to position the part within the build platform and generate the necessary support structures. Each optimised orientation, as determined in the previous step, was placed in the build platform individually to ensure a controlled evaluation of its performance during the printing process. To ensure stability and manufacturability across different part orientations, various support structures were applied based on the selected optimisation criteria. Figure 3 illustrates the support configurations used for the four first-ranked part orientations from the Siemens NX optimization process, incorporating a combination of tree supports, point supports, and perforated block & line supports. While Siemens NX facilitates support generation, the selection of support types and placement was manually determined by the user, ensuring that the support strategy aligned with printability requirements. To optimize material usage and reduce print time, perforated block and line supports were specifically applied to the part. These structures provided sufficient stability during printing while minimizing material consumption and post-processing effort. Additionally, the part was positioned with a 3 mm offset from the build plate to facilitate proper removal after printing. To support this offset, additional support structures were generated beneath the part to ensure adequate adhesion during the printing process while allowing for easier detachment upon completion. The build strategy was generated through Siemens NX with a 40 µm layer thickness, ensuring a high-resolution fabrication process with precise layer deposition.

### 2.4. Thermal Distortion Prediction

Atlas 3D software was used to predict thermal distortions in the selected part orientations obtained from Siemens NX. The software employs a computational model that simulates the thermal stresses and warping tendencies of the part based on factors such as material properties, laser scanning strategy, and layer thickness. For each Siemens NX optimised orientation, the corresponding STL file was uploaded to Atlas3D, and a thermal distortion analysis was performed.

The STL file was uploaded to the Atlas 3D cloud-based platform, where a new job was created. The build parameters, including material type, layer thickness, and scanning speed, were defined according to the specifications of the selected LPBF system. Manual orientation validation was conducted by selecting the pre-determined Siemens NX orientations and running distortion simulations to assess the predicted warping effects. The support structures, generated in Siemens NX for each orientation, were imported as STL files. Once the simulations were complete, distortion results were analysed using Atlas3D’s built-in visualization tools, which provided a colorimetric distortion map indicating displacement in the X, Y, and Z directions.

### 2.5. Experimental Validation via LPBF Printing

To validate the simulation predictions and orientation optimization results, one motor bracket was printed using the Laser Powder Bed Fusion (LPBF) process. The orientation selected for experimental validation was the overheating-optimized orientation, as it provided a balanced compromise between distortion risk and thermal stability in simulation results. The part was manufactured using the LPBF technique (INTECH, SF1 iFusion150, Intech Additive Solutions Ltd., Bangalore, India) with AlSi10Mg material, employing process parameters optimised for high relative density (>99.9%) specific to the selected material. These parameters were determined based on prior process development and calibration, ensuring consistent mechanical properties and minimal internal porosity. The part then underwent a standard stress-relief heat treatment with a ramp rate of 5 °C/min, a soak time of 2 h at 300 °C, and air cooling. After heat treatment, the support structures were removed manually

### 2.6. Distortion Measurement and Comparison

To evaluate the actual geometric distortion of the printed motor bracket and validate the predictions generated by Atlas 3D, the printed motor bracket was scanned at two distinct stages of the post-build workflow. First, the component was scanned on the build platform, with support structures intact and prior to any post-processing. This scan was used to assess the geometric deviations corresponding to Atlas 3D’s prediction of distortion due to the build process, that is, the deformation occurring between the nominal STL and the as-built part, primarily reflecting thermally induced warping during fabrication. Specifically, this comparison focused on the displacement normal to the part surface, which offers a sensitive measure of surface deviation due to residual thermal gradients and constrained shrinkage during layer-by-layer construction.

Following this initial scan, the part underwent stress-relief heat treatment and subsequent support removal. A second scan of the fully post-processed component was then performed. This scan enabled comparison with Atlas 3D’s prediction of total distortion, which accounts for both the build-induced warping and the additional shape change caused by residual stress relaxation after support removal. Again, the deviation was evaluated in terms of displacement normal to the part surface, as this parameter directly captures dimensional inaccuracies relevant to functional fit and tolerance verification.

For the scan conducted on the build platform, a single scan was captured using the EinScan HX laser scanner (SHINING 3D, Hangzhou, China), which provides a single-shot accuracy of ±0.04 mm and a volumetric accuracy of 0.04 mm + 0.06 mm/m, to record the geometry of the printed motor bracket with all support structures intact. The total expected measurement uncertainty was approximately ±0.046 mm. Given the accessibility of the external surfaces in this state, only one scan pass was required. The resulting mesh was directly exported from EXScan software (version 1.4.1.2; SHINING 3D, Hangzhou, China) as an STL file. This STL was then imported into Siemens NX, where a point-to-point alignment with the nominal CAD model was performed. This alignment enabled precise measurement of the geometric deviations introduced during the LPBF process and supported comparison with Atlas 3D’s prediction of build process distortion.

In contrast, for the post-processed geometry, after heat treatment and support removal, two scans were required to capture both the top and bottom surfaces of the component, ensuring complete coverage of complex and internal features. The two scans were aligned in EXScan software using the point-to-point alignment tool. The measurement uncertainty for the post-processed scans is consistent with the as-built scans, with an expected volumetric accuracy of approximately ±0.046 mm. This aligned mesh was subsequently exported as an STL and imported into Siemens NX for further refinement. Within NX’s reverse engineering module, surface defects such as holes and open edges were repaired to produce a watertight mesh suitable for analysis. The processed scan was then aligned with the original CAD model using a point set to point set & best fit alignment, enabling an accurate surface deviation analysis. The resulting comparison map provided a visual and quantitative representation of the total distortion, including both build-induced and post-processing deformation, and served as a critical input for validating Atlas 3D’s simulation of full-process thermal distortion.

### 2.7. Sustainability Assessment

The life cycle assessment (LCA) [23] of Laser Powder Bed Fusion (LPBF) was conducted in accordance with ISO 14040 [24] and ISO 14044 [25], following the carbon footprint principles of ISO 14067 [26]. The goal was to quantify greenhouse gas emissions for LPBF part production to support sustainability evaluation. The functional unit was defined as one finished part, which served as the basis for all reported results. Two system boundaries were assessed: (i) gate-to-gate, covering machine energy during build and auxiliary phases, shielding gas use, powder handling and reuse, and post-processing operations such as heat treatment and optional machining/finishing; and (ii) cradle-to-gate, which additionally includes upstream processes such as ingot production, intermediate forming (e.g., rolling, wire drawing), and powder atomisation [27,28]. Emission factors were sourced from the literature [29] and supplemented with regional electricity mix data for EU-27 (2023). Specifically, electricity-related emissions were modelled using the EU-27 (2023) average energy mix, with a carbon intensity of approximately 207 g CO_2_e/kWh (electric), reflecting a representative industrial context and highlighting the influence of regional energy systems on sustainability outcomes [30]. This corresponds to about 99 g CO_2_e/kWh (primary energy, “oe”), where “el” denotes the delivered electricity, while “oe” represents the upstream primary energy equivalent, accounting for generation, transmission, and distribution losses [31,32]. For methodological consistency, a Primary Energy Factor (PEF) of 2.1 was applied in line with EU guidance (2018) to convert final electricity consumption into primary energy demand [33]. Activity data were derived from part geometry, build parameters, and process settings. Allocation followed a cut-off approach for material recovery, while shared energy loads (e.g., heat treatment) were allocated by mass. Assumptions included a powder recycling rate of 95%, inclusion of stress-relief heat treatment, and exclusion of end-of-life. Emissions were calculated by multiplying activity data with corresponding emission factors. The total carbon footprint was computed as:

(1) $${CO}_{2e_{total}}=\;\sum_{j}A_{j\;}\times EF_{j}$$

where *A_j_* represents activity data (e.g., kWh of electricity, m^3^ of shielding gas, kg of virgin powder) and *EF_j_* the corresponding emission factor. Activity data were derived from deterministic relationships between part geometry, build rate, layer height, and process parameters.

The additive manufacturing route consumed approximately 5.6 kg of material and ~16 kWh of electricity per build. Metal scrap generated during the process amounted to around 30 g, of which roughly 13 g were directed into recycling streams. Additional resource inputs included argon gas, compressed air, and water. Energy use was calculated at the part level and covered all key stages: printing, post-processing, and powder handling. Upstream data used in the cradle-to-gate modelling included electricity consumption of 17 kWh/kg and water consumption of 36 kg/kg for ingot production. For the gas atomisation process, an argon consumption of 3 m^3^/kg and electricity use of 6 kWh/kg were applied. Contributions from support structure reuse and powder recovery were also accounted for via energy credits, which improved the environmental score for build orientations with simpler geometries or reduced support requirements.

Environmental performance for each build orientation was evaluated using a resource- and energy-based model. This model combines material composition data with eco-database values for embodied energy and CO_2_ emissions, accounting for both virgin and recycled content. Recycling and reuse practices, including recovery of unmelted powder and recycling of support structures, were incorporated by assigning recycling credits, thereby reflecting closed-loop material practices. Energy consumption was calculated at the part level and included printing, post-processing, and powder handling stages. Contributions from support and powder recycling were also incorporated, providing energy credits that improved the environmental score for orientations with simpler geometries or reduced support needs.

### 2.8. Cost Assessment

A detailed cost analysis was conducted to evaluate the impact of different build orientations—minimised support volume, reduced build time, overheating control, and minimised surface area—on the overall cost of manufacturing a topology optimised bracket via Laser Powder Bed Fusion (LPBF). The analysis followed a parametric cost model that includes all relevant stages from material input to post-processing. Each orientation, previously selected through Siemens NX, was imported into the cost model to generate orientation-specific cost outcomes.

The total manufacturing cost for each orientation was calculated using a structured cost breakdown that incorporates material usage, process times, labour, machine operation, and energy consumption. The total cost was computed according to the following expression:

Total Cost = CM + CDP + CIS + CBS + CD + CR + CC + CU + CS + CP(2)

where

- Material Cost (CM) → CM = Material Cost per kg × Mass of the part
- Data Preparation Cost (CDP) → CDP = Data Preparation Time × Engineer Hourly Rate
- Machine Setup Cost (MSC) → MSC = Time × (Machine Hourly Rate + Labor Rate)
- Laser Melting Cost (LMC) (Deposition Cost (CD)) → LMC = Deposition Time × Machine Hourly Rate
- Recoating Cost (CR) → CR = Recoating Time × Machine Hourly Rate
- Cooldown Cost (CC) → CC = Cooldown Time × Machine Hourly Rate
- Unloading Cost (CU) → CU = Unloading Time × (Machine Hourly Rate + Labor Rate)
- Separation & Post-processing Cost (CS) → CS = Separation Time × (Labor Rate + System Cost)
- Other Process Costs (CP) → CP = Total Process Time × Energy Cost per Hour + Consumables Cost
- Support removal (SR) → SR = Time × Staff Salary per hour

## 3. Results

### 3.1. Simulation-Based Orientation Optimisation

**Figure 4:**
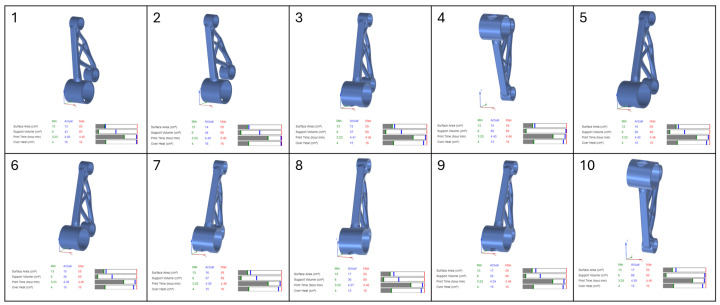
Optimised orientations ranked based on surface area minimization in Siemens NX. The selected orientations reduce the total supported surface area, minimising post-processing requirements.

**Figure 5:**
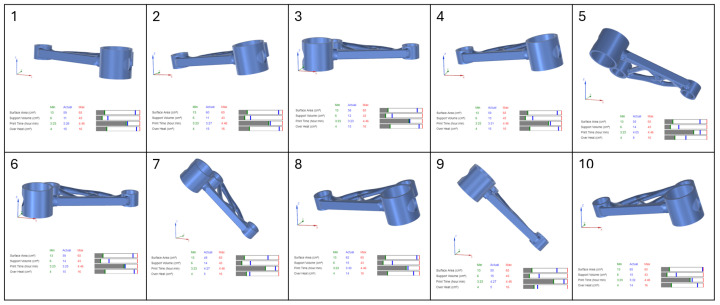
Optimised orientations ranked based on support volume minimization in Siemens NX. These orientations prioritise reducing the amount of support material required, which can decrease material usage and post-processing efforts.

**Figure 6:**
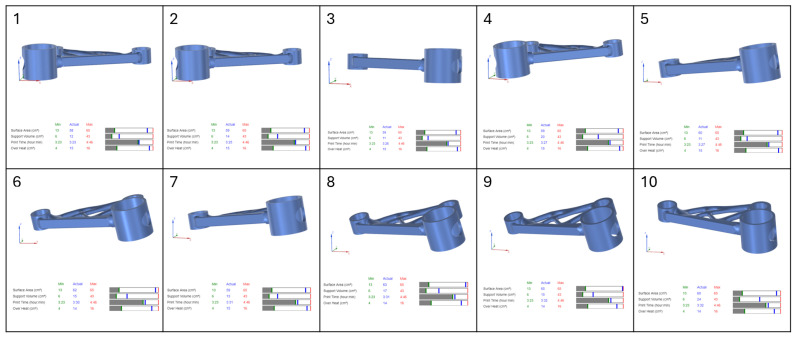
Optimised orientations ranked based on print time minimization in Siemens NX. The chosen orientations reduce total build duration, enhancing production efficiency.

**Figure 7:**
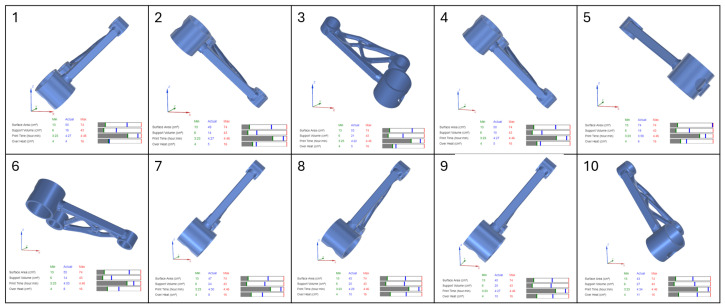
Optimised orientations ranked based on overheating minimization in Siemens NX. These orientations focus on mitigating thermal accumulation to prevent residual stress and distortion.

The results of the part orientation optimization conducted in Siemens NX demonstrate the impact of different build orientations on surface area, support volume, print time, and overheating. Each optimization run was performed by assigning a 100% weighting to a single criterion, while the remaining parameters were set to 0%, ensuring that each run focused exclusively on one objective. The software generated 10 ranked orientations per optimization run. The results, displayed in Figure 4, Figure 5, Figure 6 and Figure 7, include the 3D representations of each orientation and their corresponding numerical evaluations for the four optimization criteria. The bar charts beneath each orientation visually compare the actual values with the minimum and maximum values recorded across all possible orientations.

In Figure 4, which presents the surface area optimization, the goal was to minimise the amount of part surface requiring support structures, thereby reducing post-processing efforts and material waste. The bar charts indicate that for orientations with lower supported surface areas, the blue marker (actual value) is positioned close to the green line (minimum value) in the surface area bar. However, in these same orientations, the blue marker shifts toward higher values in the support volume and print time bars, indicating an increase in these parameters. This suggests that orientations with minimal support surface do not necessarily yield the lowest support material consumption or the fastest print times. Overheating remains relatively stable across different orientations, as seen in the corresponding overheating bar.

For support volume optimization, shown in Figure 5, the objective was to reduce the amount of material required for supports, which directly impacts material usage and cost. The bar charts reveal that orientations with low support volumes have their blue markers aligned with the green minimum values, confirming their effectiveness in minimising support structures. However, print time increases in some cases, as indicated by a blue marker positioned farther from the green minimum and closer to the red maximum boundary. This highlights a key trade-off, where orientations that reduce support volume may extend build times due to modified part positioning and scanning strategies. Overheating values fluctuate across orientations, indicating that reducing support volume does not necessarily correlate with improved thermal stability.

In Figure 6, which illustrates the print time optimization, the primary focus was on minimising the total build duration to improve production efficiency. The bar charts show that orientations with shorter print times have their blue markers aligned with the green minimum in the print time category. However, the support volume and surface area bars show higher blue markers, meaning that while print time is reduced, support material requirements increase. This suggests that the fastest printing orientations often require additional support structures, potentially offsetting efficiency gains by increasing material consumption and post-processing effort. The overheating bars display moderate fluctuations across different orientations, with some configurations maintaining stable thermal conditions while others indicate a slight increase in thermal accumulation.

The final optimization, presented in Figure 7, focused on minimising overheating, aiming to reduce thermal accumulation and mitigate residual stresses. The bar charts confirm that overheating values were successfully reduced, as indicated by the blue markers positioned near the green minimum in the overheating bar. However, many of these orientations exhibited increased support volume and print time, reinforcing the trade-off between thermal stability and manufacturing efficiency. Some orientations that effectively minimised overheating did so at the cost of increased material usage, which could be undesirable in scenarios where support volume is a limiting factor.

The analysis of the bar charts across all optimization cases highlights the complex interactions between the four key parameters. While Siemens NX successfully identifies the best orientations for individual objectives, the trade-offs observed in the bar charts suggest that a multi-objective approach is necessary for a balanced selection. In practical applications, an optimal orientation must be chosen based on the prioritization of competing factors, as improvements in one area may negatively affect another.

### 3.2. Effect of Orientation Optimisation on Build Preparation Parameters

The influence of part orientation optimisation on build preparation parameters was evaluated based on key factors such as print time, support volume, total number of layers, and maximum print height. These parameters varied depending on the selected optimisation objective, as shown in Table 1.

The Surface Area optimised orientation resulted in the highest number of layers (2787) and tallest print height (111.48 mm) but had a relatively low support volume (368.89 mm^3^), leading to a moderate print time of 4.68 h. This strategy effectively minimised the amount of part surface requiring supports but required a taller build, impacting layer count. In contrast, the Support Volume optimised orientation significantly reduced support material usage (1366.93 mm^3^), leading to a shorter build height (31.88 mm) and a print time of 3.57 h. This confirms that prioritising support volume reduction effectively decreases material consumption, though layer count remained relatively high (797 layers). The Print Time optimised orientation had the lowest number of layers (725) and a minimal print height (29 mm) but required a higher support volume (1518.7 mm^3^). While this orientation reduced total build time to 3.53 h, it increased material usage due to additional support structures, which could impact post-processing efforts. Lastly, the Overheating optimised orientation sought to minimise thermal accumulation while maintaining structural integrity. This approach resulted in a relatively high layer count (2324 layers) and print height (92.96 mm) while keeping support material volume moderate (439.01 mm^3^). The final print time was 4.42 h, balancing thermal management with manufacturability.

These results demonstrate the relationship between part orientation optimisation and key build parameters, highlighting important trade-offs. While Support Volume Optimisation effectively minimised support material usage, it resulted in a lower print height but a higher number of layers, affecting scanning time. Print Time Optimisation significantly reduced the number of layers, leading to a shorter build duration, but required increased support volume to ensure part stability. Surface Area Optimization, on the other hand, increased build height to reduce supported surface area, which impacted total print time. Lastly, Overheating Optimisation aimed to balance thermal stability and manufacturability, leading to moderate print times and support usage. These findings emphasize the importance of considering both material efficiency and thermal effects when selecting part orientations to achieve optimal printability in additive manufacturing.

### 3.3. Effect of Orientation Optimisation on Thermal Distortion

**Figure 8:**
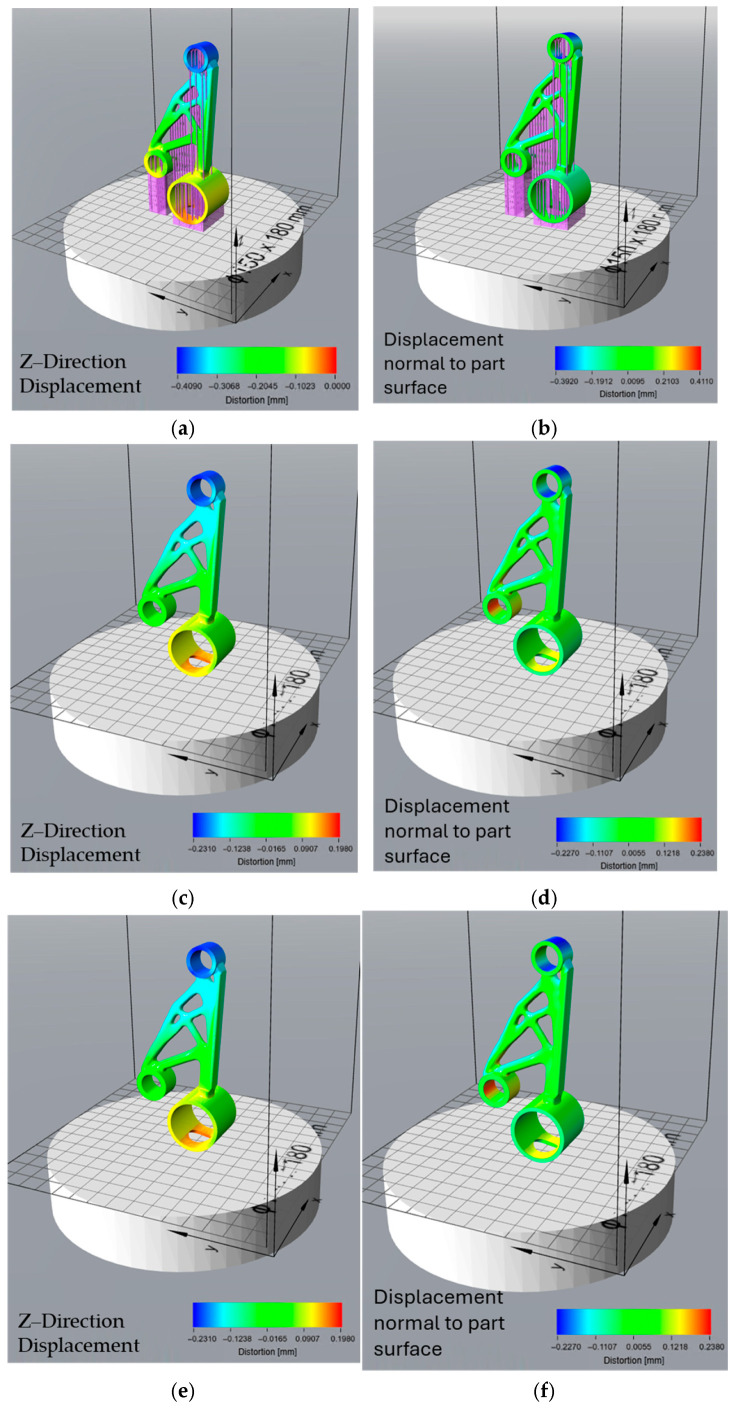
Distortion analysis for the Surface Area-Optimized Orientation from Atlas 3D. (**a**,**b**) show distortion due to the build process (as-built to STL) with Z-direction displacement and displacement normal to the part surface, respectively. (**c**,**d**) represent distortion due to residual stress (post-support removal to as-built part), showing displacement in the Z-direction and normal to the part surface. (**e**,**f**) illustrate total distortion (post-support removal to STL), combining both build process and residual stress distortions, represented in Z-direction displacement and displacement normal to the part surface.

The surface area-optimized orientation exhibited significant distortion during the build process (Figure 8a,b), with a maximum displacement of −0.409 mm in the Z-direction. This orientation, which aims to minimize the contact area with the build plate, resulted in higher thermal stresses, leading to pronounced warping and shrinkage in the vertical direction. The displacement normal to the part surface ranged from −0.392 mm to 0.411 mm, indicating substantial surface deformation. After support removal (Figure 8c,d), the residual stress-induced distortion was relatively moderate, with Z-direction displacement values between −0.231 mm and 0.198 mm. The total distortion (Figure 8e,f), combining the build process and support removal effects, ranged from −0.227 mm to 0.238 mm, suggesting that while initial warping was significant, the final part deformation remained within a moderate range.

**Figure 9:**
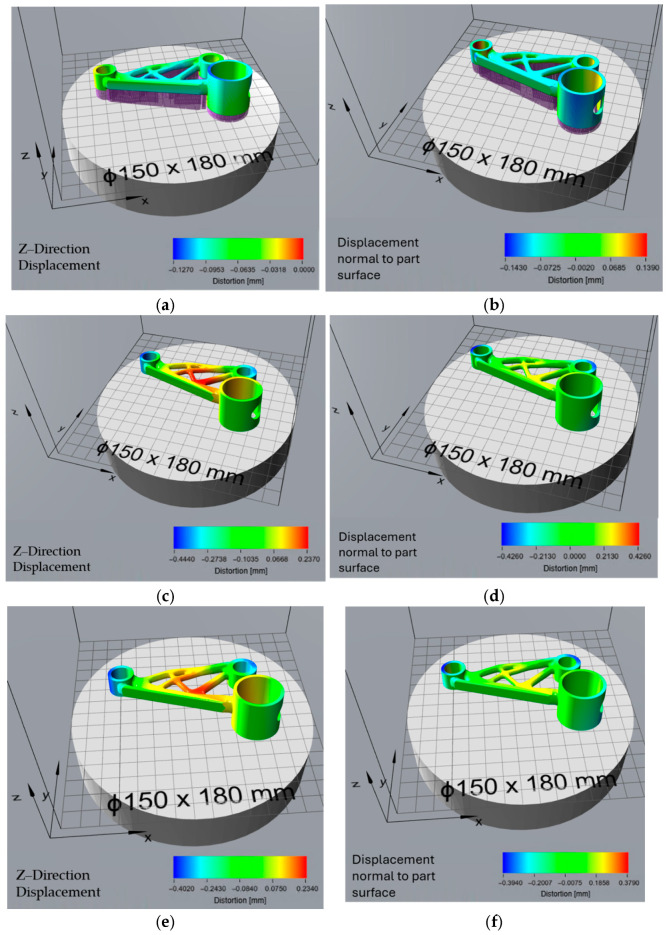
Distortion analysis for the Support Volume-Optimized Orientation from Atlas 3D. (**a**,**b**) illustrate distortion due to the build process (as-built to STL), with (**a**) showing Z-direction displacement and (**b**) depicting displacement normal to the part surface. (**c**,**d**) represent distortion due to residual stress (post-support removal to as-built part), highlighting displacement in the Z-direction and normal to the part surface, respectively. (**e**,**f**) demonstrate total distortion (post-support removal to STL), combining both build process and residual stress distortions, represented in Z-direction displacement and displacement normal to the part surface.

The support volume-optimized orientation, designed to reduce the material required for support structures, demonstrated the lowest distortion during the build process (Figure 9a,b), with a Z-direction displacement ranging from −0.127 mm to 0 mm. The displacement normal to the part surface also showed minimal variation, ranging from −0.143 mm to 0.139 mm, suggesting that this orientation effectively minimized surface warping. However, after support removal (Figure 9c,d), the part exhibited higher residual stress relaxation, leading to an increased Z-direction displacement of −0.444 mm to 0.237 mm. This indicates that while support structures effectively constrained the part during printing, their removal led to greater deformation. The total distortion (Figure 9e,f) values ranged from −0.402 mm to 0.234 mm, showing that although the build process distortion was minimal, significant shape changes occurred after support removal.

**Figure 10:**
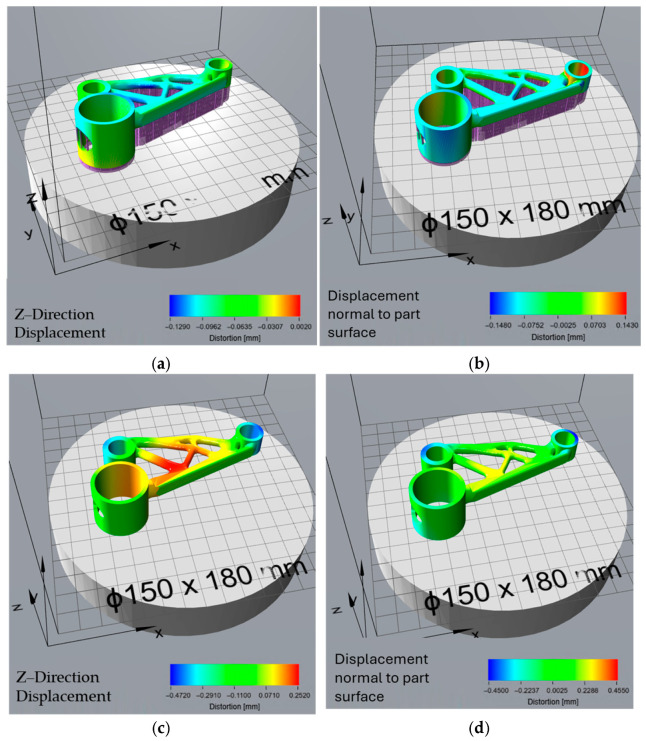

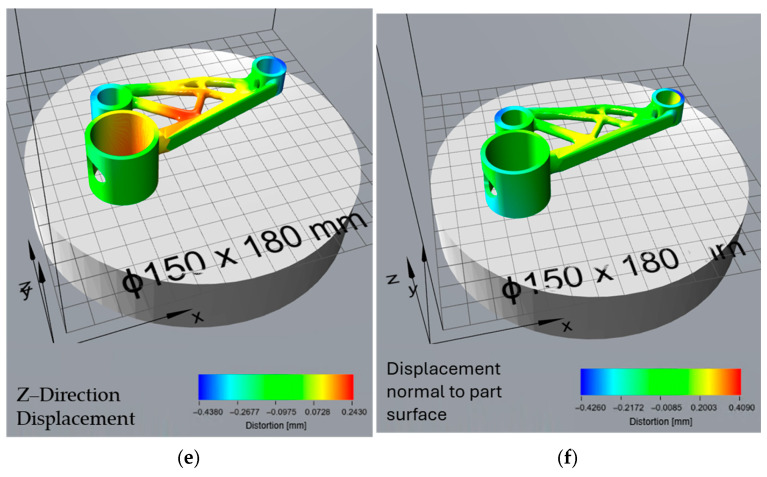
Distortion analysis for the Print Time-Optimized Orientation from Atlas 3D. (**a**,**b**) show distortion due to the build process (as-built to STL), with (**a**) representing Z-direction displacement and (**b**) displaying displacement normal to the part surface. (**c**,**d**) illustrate distortion due to residual stress (post-support removal to as-built part), with (**c**) showing Z-direction displacement and (**d**) depicting displacement normal to the part surface. (**e**,**f**) represent total distortion (post-support removal to STL), incorporating both build process and residual stress effects, with (**e**) showing Z-direction displacement and (**f**) illustrating displacement normal to the part surface.

The print time-optimized orientation, aimed at reducing manufacturing duration, resulted in minimal build process distortion (Figure 10a,b), with Z-direction displacement values between −0.129 mm and 0.002 mm. The displacement normal to the part surface was similarly controlled, ranging from −0.148 mm to 0.143 mm. However, after support removal (Figure 10c,d), this orientation exhibited the highest residual stress-induced distortion among all cases, with a Z-direction displacement of −0.472 mm to 0.252 mm. This suggests that while the chosen orientation allowed for efficient printing, it also accumulated high residual stresses, leading to significant post-processing deformations. The total distortion values (Figure 10e,f) were among the highest, with Z-direction displacements between −0.438 mm and 0.243 mm, as well as displacement normal to the part surface ranging from −0.426 mm to 0.409 mm, indicating considerable geometric deviations in the final part.

**Figure 11:**
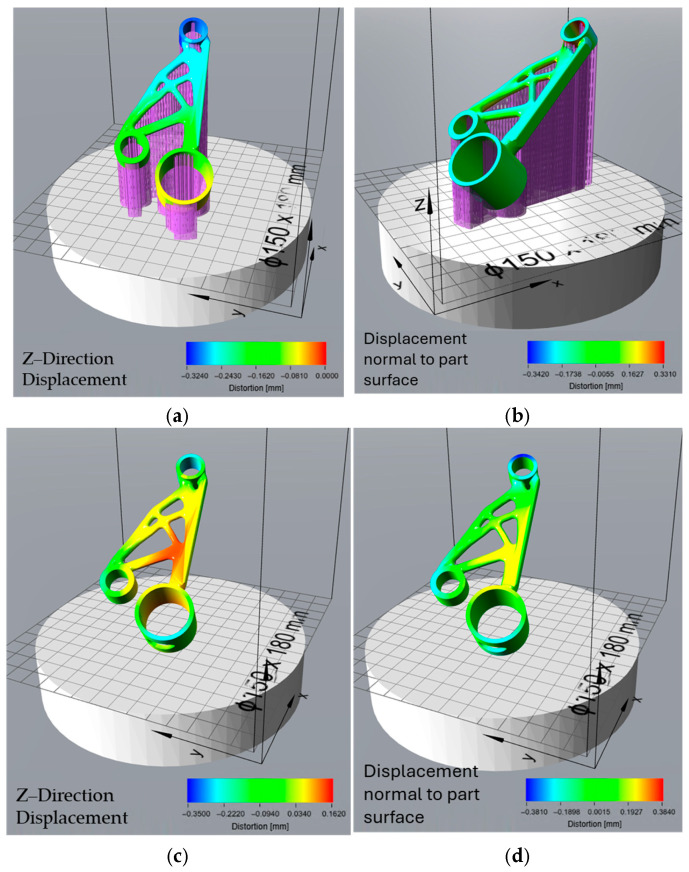

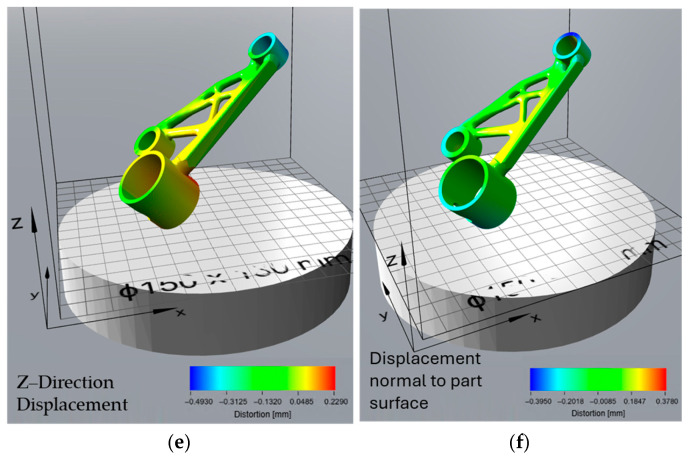
Distortion analysis for the Overheating-Optimized Orientation from Atlas 3D. (**a**,**b**) depict distortion due to the build process (as-built to STL), where (**a**) shows Z-direction displacement and (**b**) represents displacement normal to the part surface. (**c**,**d**) illustrate distortion due to residual stress (post-support removal to as-built part), with (**c**) highlighting Z-direction displacement and (**d**) displaying displacement normal to the part surface. (**e**,**f**) represent total distortion (post-support removal to STL), combining both build process and residual stress effects, with (**e**) showing Z-direction displacement and (**f**) illustrating displacement normal to the part surface.

The overheating-optimized orientation, designed to mitigate excessive thermal accumulation, resulted in moderate build process distortion (Figure 11a,b), with a Z-direction displacement between −0.324 mm and 0 mm. The displacement normal to the part surface ranged from −0.342 mm to 0.331 mm, indicating moderate surface warping. After support removal (Figure 11c,d), the residual stress-induced distortions were lower than those in the print time and support volume orientations but higher than the surface area-optimized orientation, with Z-direction displacement values between −0.35 mm and 0.162 mm. The total distortion values (Figure 11e,f), however, were the most severe, with a Z-direction displacement of −0.493 mm to 0.229 mm, making this the least dimensionally accurate orientation. Despite its ability to mitigate overheating, this configuration led to the highest overall shape deviation, suggesting a trade-off between thermal management and geometric stability.

### 3.4. Deviation Analysis of As-Built and Post-Processed Geometry

**Figure 12:**
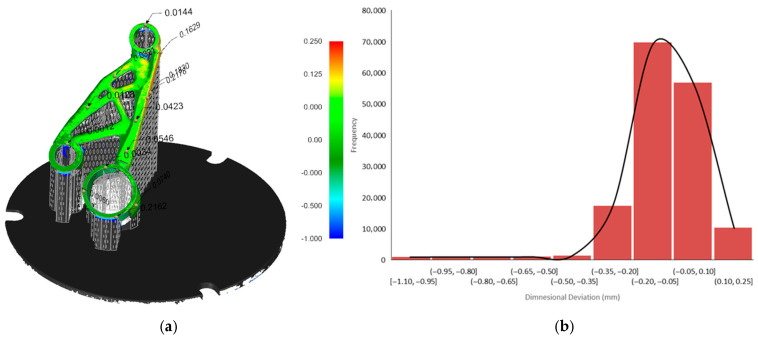
(**a**) Deviation map of the motor bracket in the as-built condition, scanned directly on the build platform with support structures intact. Color gradients indicate the magnitude of surface deviation relative to the nominal CAD geometry, with green representing minimal deviation and blue/red representing negative and positive deviations, respectively. (**b**) Histogram of dimensional deviation values across 156,668 measured surface points.

For the as-built condition (pre-support removal), a total of 156,668 surface points were evaluated. The deviation map (Figure 12a) revealed that the majority of the part’s surface exhibited only minor distortion, with most deviations falling between −0.2 mm and +0.1 mm, as shown in the corresponding histogram (Figure 12b). The maximum positive deviation reached +0.249 mm, while the maximum negative deviation was −1.099 mm, the latter occurring in localized regions—likely around unsupported overhangs or thin truss-like features. The average deviation across the surface was −0.093 mm, indicating a slight overall contraction relative to the nominal geometry. The standard deviation of 0.168 mm reflects the variation in distortion across the surface. Detailed statistical parameters summarising these deviations are provided in Table 2.

**Figure 13:**
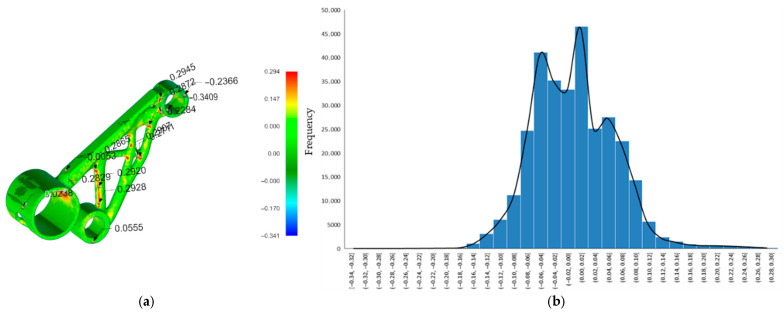
(**a**) Deviation map of the motor bracket after heat treatment and support removal, showing geometric deviation relative to the nominal CAD geometry. The colour scale indicates the magnitude of deviation. (**b**) Histogram of dimensional deviations measured across 298,408 surface points.

Following heat treatment and mechanical removal of the support structures, this second analysis (Figure 13a) included 305,284 surface points, providing higher spatial resolution. The maximum deviation was +0.294 mm, and the minimum deviation was −0.341 mm, indicating a substantial reduction in geometric deviation range compared to the pre-processed scan. The average deviation dropped to just −0.0015 mm, and the standard deviation was also significantly reduced to 0.062 mm. As observed in the histogram (Figure 13b), the majority of deviations clustered tightly within a narrow band between 0.00 mm and +0.02 mm, highlighting the geometric stabilisation achieved through heat treatment and the release of residual stresses. Detailed statistical parameters summarising these deviations are provided in Table 3.

### 3.5. Sustainability Assessment

**Figure 14:**
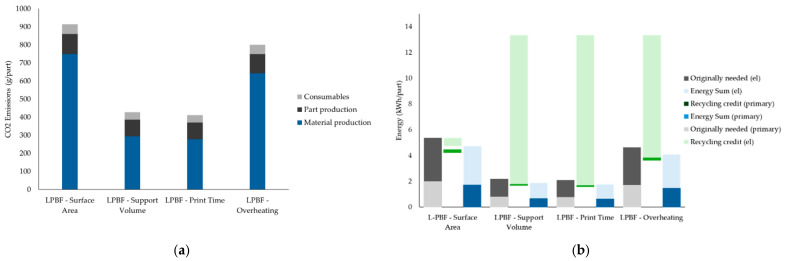
Comparison of environmental performance across build orientations in LPBF: CO_2_ emissions (**a**) and embedded energy consumption (**b**) per part for different part orientation strategies.

The sustainability assessment evaluated the environmental impacts associated with different part orientation strategies optimised in Siemens NX, focusing on CO_2_ emissions (g/part) and embedded energy consumption (kWh/part), as shown in Figure 14. Among the evaluated strategies, orientations that minimised support volume and print time exhibited the lowest environmental impacts, whereas surface area minimisation resulted in the highest. Specifically, the support volume and print time strategies achieved CO_2_ emissions of approximately 400–420 g/part, while surface area minimisation exceeded 900 g/part, primarily due to greater material usage and extended processing times (Figure 14a). Material production accounted for the largest share of total emissions across all cases, followed by part production and consumables. Embedded energy analysis (Figure 14b) confirmed similar trends, with support volume and print time optimisations resulting in total energy consumptions of approximately 2 kWh/part, compared to nearly 5 kWh/part for surface area minimisation. Recycling credits contributed significantly to reducing net energy consumption, especially for support volume and print time optimised orientations.

### 3.6. Effect of Orientation Optimisation on Manufacturing Cost

**Figure 15:**
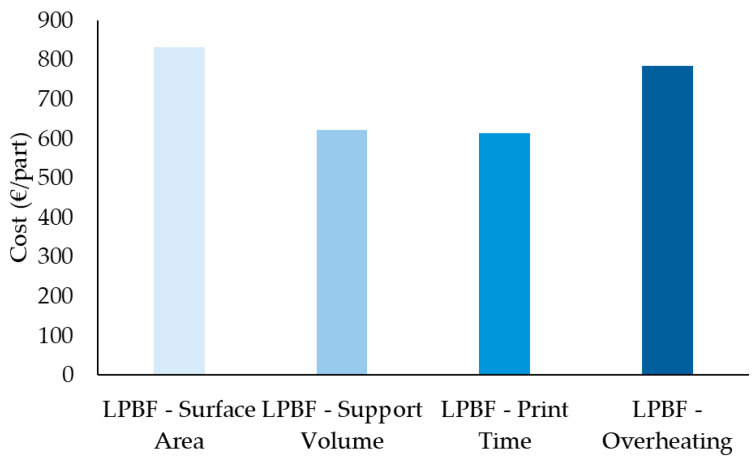
Manufacturing cost per part for different LPBF part orientation strategies, including surface area minimisation, support volume minimisation, print time reduction, and overheating control.

The cost assessment evaluated the manufacturing cost per part for the different LPBF orientation strategies optimised in Siemens NX, as shown in Figure 15. The results demonstrate that orientations minimising support volume and print time resulted in the lowest total costs, at 621.78 €/part and 613.88 €/part, respectively, whereas surface area minimisation and overheating control strategies incurred significantly higher costs of 832.34 €/part and 785 €/part, respectively. The elevated cost associated with surface area minimisation is primarily attributed to increased material consumption, reflected in the higher cost per unit volume (63.25 €/cm^3^), while the overheating control strategy also showed increased cost per unit volume (59.65 €/cm^3^).

## 4. Discussion

Our results reveal a consistent tension between build-stage stability and post-processing accuracy: orientations that are very stable on the plate (typically those optimising print time or support volume) tend to undergo greater deformation after support removal as residual stresses relax, whereas orientations that tolerate more on-plate distortion (such as minimised supported surface area) often yield a more stable final geometry. At the same time, thermal uniformity does not automatically translate into geometric fidelity. Reducing local overheating lowers peak thermal gradients, yet total shape deviation can still be substantial unless the chosen orientation is paired with targeted compensation, for example, local stiffening, selective support densification, or pre-deformation. A further measurement insight is that assessing normal-to-surface deviation, the offset measured perpendicular to the CAD surface at each point, captures distortion on sloped and curved features that Z-only measurements can miss. In this study, this metric also aligned well with the predictive patterns of reduced-order simulation when experimentally validated against scans. Finally, the sustainability results show a practical alignment between efficiency and environmental performance: orientations that reduce build height and support requirements consistently cut energy use, CO_2_ emissions, and cost, offering a clear route to reconcile manufacturability with sustainability goals.

In the comparison of the four different orientation strategies (minimised surface area, minimised support volume, minimised print time, and overheating control), each optimisation objective influenced distortion mechanisms differently owing to changes in thermal gradients, the effectiveness of support constraints, and residual-stress relief.

The orientations generated in Siemens NX were optimized based on specific criteria—minimizing surface area, reducing support volume, decreasing print time, and avoiding overheating. These orientations were then analysed in Atlas 3D to predict thermal distortions and determine the extent of deformation resulting from both the build process and residual stress relaxation after support removal. The results from the distortion analysis provide insights into the impact of different orientation strategies on the geometric accuracy and stability of the printed part. Each optimization criterion—surface area, support volume, print time, and overheating—resulted in distinct distortion patterns due to varying thermal stresses, support configurations, and residual stress relief mechanisms. The choice of orientation directly influenced the magnitude and type of distortions observed throughout the build process, post-support removal, and final shape deviation.

Among all orientations, the surface area optimization exhibited the highest distortion during the build process, with a maximum Z-direction displacement of −0.409 mm. This significant deviation suggests that minimizing contact with the build plate leads to increased thermal stress accumulation, which manifests as severe warping and shrinkage in the vertical direction. However, after support removal, the residual stress-induced distortion was relatively lower than in other orientations, with Z-direction displacement ranging from −0.231 mm to 0.198 mm. This indicates that while initial thermal stresses were high, the support structures effectively constrained much of the stress-induced movement, resulting in moderate final part distortion with total displacement values between −0.227 mm and 0.238 mm.

The support volume optimization yielded the lowest distortion during the build process, with Z-direction displacement values between −0.127 mm and 0 mm. This suggests that reducing support structures did not compromise initial print stability. The displacement normal to the part surface was also minimal, ranging from −0.143 mm to 0.139 mm, further confirming that this orientation effectively mitigated early-stage warping. However, after support removal, the residual stress relaxation was significantly higher than in other cases, resulting in a maximum Z-direction displacement of −0.444 mm. This highlights a key drawback of reducing support structures: although the part remains stable during printing, it experiences greater stress relief deformation once the supports are removed. These results are consistent with the findings of Zongo et al., who also observed that reducing support structures in LPBF builds led to greater distortion after support removal [34]. Their study emphasized that while minimal supports improve build efficiency, they allow more residual stress to relax post-processing, leading to dimensional inaccuracies, supporting the critical trade-off identified in the present work. The total distortion was still moderate compared to other orientations, with values ranging from −0.402 mm to 0.234 mm, making this orientation a viable trade-off between minimizing support usage and maintaining dimensional accuracy. This outcome aligns with Zhang et al., who observed that orientations optimized for minimal support usage exhibited increased post-processing distortion due to stress redistribution after support removal [35].

The print time optimization aimed to reduce manufacturing duration and exhibited similar low distortion values during the build process, with a Z-direction displacement of −0.129 mm to 0.002 mm. The displacement normal to the part surface also remained relatively controlled, within the range of −0.148 mm to 0.143 mm. However, this orientation suffered from the highest residual stress-induced deformation after support removal, with Z-direction displacement values reaching −0.472 mm. This indicates that while printing was efficient, significant residual stresses accumulated due to the rapid thermal cycles and material solidification patterns. The total distortion values, ranging from −0.438 mm to 0.243 mm, were among the highest, suggesting that while reducing print time is beneficial for efficiency, it introduces substantial geometric deviations that may require extensive post-processing. Similarly, Zhang et al. reported that while minimizing build time improves productivity, it often leads to thermal instability and increased distortion risk, particularly for geometrically complex parts [35].

The overheating-optimized orientation was selected to mitigate excessive thermal accumulation, but it resulted in considerable shape distortion. The Z-direction displacement during the build process was −0.324 mm to 0 mm, which is lower than the surface area optimization but higher than the support volume and print time orientations. The displacement normal to the part surface ranged from −0.342 mm to 0.331 mm, indicating moderate surface deformation. After support removal, residual stress relaxation was observed, with Z-direction displacement values of −0.35 mm to 0.162 mm, which was lower than the print time and support volume orientations but still significant. However, the total distortion was the highest among all orientations, with a Z-direction displacement of −0.493 mm to 0.229 mm. This suggests that while managing overheating is crucial for material integrity, it comes at the cost of increased shape deviation, necessitating additional compensation strategies.

A detailed comparison of the different optimization criteria is presented in Table 4. These findings highlight the trade-offs between minimizing distortion during the build process and controlling shape deviations after support removal.

From the comparative analysis, it is evident that the support volume and print time optimizations resulted in the lowest initial build process distortions, making them the most stable orientations during printing. However, they also exhibited the highest residual stress-induced deformations, highlighting a critical trade-off between initial stability and post-processing distortions. The surface area optimization experienced the highest warping during the build process, but the final distortion was more controlled after support removal, making it a reasonable option for minimizing stress relief-induced shape deviations. The overheating optimisation, while minimising thermal accumulation, resulted in distortions, underscoring that reduced heat build-up does not necessarily correspond to improved dimensional accuracy.

Ultimately, the choice of orientation depends on the primary objective of the print. If minimizing build process distortion is the priority, the support volume or print time optimization is preferable. This orientation-dependent trade-off is shown in the work of Qin et al., who developed an automated framework for selecting build orientation based on multiple objectives, including surface quality, support volume, and distortion risk [36]. Their findings similarly illustrate that orientations optimized for reduced build time may incur greater deformation due to thermal stress, supporting the conclusion that distortion control must be integrated into early design-stage orientation decisions. However, if reducing residual stress-induced deformations is more critical, the surface area optimization presents a viable option. For applications where overheating is a major concern, additional compensation strategies should be employed to counteract the significant shape deviations observed in the final part. These findings emphasize the need for a holistic approach when selecting an optimal orientation, balancing printability, thermal stress management, and final geometric accuracy.

The comparison between the 3D scan of the as-printed motor bracket and the Atlas 3D prediction of build process distortion (Figure 11b) provides valuable insight into how the part’s surface geometry changes during the LPBF process due to thermal stresses. This comparison focuses on the actual surface deviation, measured in the direction normal to the surface. As a result, it captures not only vertical shrinkage or uplift but also distortion on sloped, curved, or angled surfaces, which are often overlooked in simple Z-direction analyses. Evaluating this surface-based deviation is important for understanding the overall shape accuracy, especially for topology-optimised geometries with complex curves and internal features. While Atlas 3D predicted key regions of distortion correctly, the actual scan showed larger deviations in some areas, confirming that true surface-level measurements are essential for identifying critical build errors that may affect mechanical performance or fit. The comparison between the post-processed scan (after heat treatment and support removal) and the total distortion prediction from Atlas 3D (Figure 11f) offers an even more accurate and practical view of final part quality. This prediction also uses normal surface deviation, making it suitable for full 3D shape comparison. It is the most realistic measure of real-world geometric error, accounting for both build-stage distortion and stress relief effects after support removal. This type of analysis is especially useful for quality control, as it reflects the final geometry delivered to the end user. The close match between Atlas 3D’s prediction and the scan after post-processing suggests that the simulation tool is reliable for predicting final part shape, particularly when used in combination with a 3D scanning workflow. This kind of comparison can also support more informed post-processing decisions, such as the need for machining or rework on specific features. These findings are consistent with those of Giubilini et al., who validated the predictive accuracy of Amphyon simulations by demonstrating good agreement with 3D scan results and emphasized the potential of virtual pre-compensation in improving shape fidelity [37]. Similarly, another study noted that simulation accuracy in predicting distortion strongly depends on software calibration and geometric complexity, reinforcing the need for validated workflows [7].

The histogram of the as-printed scan (Figure 12b) supports these findings by showing that most of the part’s surface points fall within a deviation range of −0.2 mm to +0.1 mm, meaning the majority of the part is relatively close to the CAD model. However, there is a noticeable skew toward negative deviation values, with a long tail extending to −1.099 mm. This indicates that while most regions are only slightly distorted, specific unsupported or thermally stressed zones, such as thin walls or overhangs, experience significant inward warping or collapse. These extreme deviations could lead to functional problems, such as poor fit during assembly or loss of dimensional tolerance, especially for mating features. By comparison, the post-processed scan histogram (Figure 13b) shows a much tighter distribution. Most surface deviations fall between 0.00 mm and +0.02 mm, with no extreme values on either side. The average deviation drops to almost zero (−0.0015 mm), and the standard deviation is reduced to 0.062 mm, compared to 0.168 mm in the as-built state. This reduction in both the range and variability of deviations confirms that stress-relief heat treatment and support removal significantly improve dimensional accuracy. Importantly, the scan also validates that Atlas 3D’s total distortion prediction (Figure 11f) is well-aligned with the final printed part, indicating that the software is more accurate when all process stages are taken into account.

These results highlight that normal surface deviation analysis, used in both the Siemens NX comparisons and Atlas 3D’s full-process predictions, is the most appropriate method for evaluating final shape accuracy, particularly for topology-optimised or curved geometries. Unlike simple vertical (Z-axis) displacement maps, which miss deformation on angled or vertical surfaces, this method gives a complete 3D assessment. This is especially important in additive manufacturing, where geometry complexity often causes multi-directional distortion. Therefore, the approach used in this study, combining 3D scanning, normal deviation comparison, and simulation validation, provides a framework for understanding and predicting distortion throughout the LPBF workflow.

Furthermore, these comparisons can support decision-making during the design and process planning stages. For instance, if simulations indicate a high likelihood of distortion in critical regions, engineers can proactively adjust the part orientation, modify support strategies, or apply local reinforcements. The ability of Atlas 3D to reasonably predict final geometry, when validated with experimental scans, also strengthens its use as a cost-effective and time-saving tool, reducing the need for iterative physical testing and reprinting.

In addition to geometric accuracy and distortion control, the choice of part orientation significantly impacts the sustainability and economic efficiency of the LPBF process. The optimization criteria evaluated, minimizing surface area, support volume, print time, and overheating, not only influenced distortion patterns but also led to notable differences in material usage, energy consumption, and production cost, as shown in Figure 14a,b and Figure 15 and Table 5. The orientation optimized for minimum surface area resulted in the highest CO_2_ emissions, energy consumption, and overall cost. This outcome is primarily attributed to the large volume of material required to build extensive support structures and the increased energy demand due to longer print times and powder usage. Specifically, the material production stage alone accounted for the majority of emissions (~750 g CO_2_/part), highlighting the environmental burden of inefficient support strategies. Conversely, the support volume and print time orientations achieved the lowest environmental impact and cost. Both strategies minimized the need for support structures and reduced build duration, directly lowering material waste, machine energy usage, and associated costs. A similar relationship between orientation, support strategy, and process efficiency was identified by Reichwein and Kirchner, who showed that build orientation has a decisive influence on support volume, build height, and part separation potential [38]. Their cost modelling demonstrated that orientations minimizing support and maximizing packing density led to significantly lower production costs and energy consumption, consistent with the sustainability advantages observed in the support volume and print time strategies of the current study. These two options recorded nearly half the total CO_2_ emissions and energy use compared to the surface area optimization. This demonstrates the strong link between build efficiency and sustainability in metal AM processes. The overheating-optimized orientation showed a moderate profile, with lower emissions and energy use than the surface area strategy but higher than the support volume and print time cases. Interestingly, although this strategy effectively reduced localized thermal stress during printing, it did not result in meaningful energy or material savings, likely due to trade-offs in part orientation that increased overall build height or complexity. The energy analysis (Figure 14b) reveals additional insights into the role of material recycling in AM sustainability. Across all orientations, recycling credits, especially for electricity, played a substantial role in reducing the net energy demand. However, the surface area optimization remained the least energy-efficient after accounting for recycling, due to its inherently inefficient geometry and support structure requirements. Malbašic et al. further support these findings, showing that minimizing build height and support structures through orientation optimization can effectively lower energy usage and material demand in LPBF, showing the environmental and economic advantages of early design-stage decisions.

The cost analysis (Figure 15) and values in Table 5 reflect these trends. The surface area optimization was the most expensive (~€832.34/part), while support volume and print time strategies were the most cost-effective (both around €610–620/part). The cost per kilogram and per cm^3^ followed a similar trend, with surface area orientation reaching up to €23,688.31/kg and €63.25/cm^3^, compared to €17,470.82/kg and €46.65/cm^3^ for the print time orientation. This confirms that reducing print time and support structures not only enhances process sustainability but also yields significant economic benefits.

These findings support the need to integrate sustainability and cost metrics into the early design and orientation selection process. While geometric accuracy and thermal stability remain critical, ignoring environmental and economic factors can lead to suboptimal manufacturing outcomes. In practice, the support volume and print time orientations represent the most balanced trade-off, offering good dimensional performance alongside reduced environmental impact and production cost.

## 5. Conclusions

This study investigated the influence of part orientation on geometric accuracy, thermal distortion, sustainability, and cost-efficiency in LPBF using a complex motor bracket as a case study. By applying orientation optimization based on four different criteria, minimising surface area, support volume, print time, and overheating, this work demonstrated that orientation selection is a key driver of both part quality and manufacturing efficiency. Distortion analysis using Siemens NX and Atlas 3D revealed that each orientation strategy led to distinct deformation patterns during the build and post-processing phases. Minimising surface area resulted in the highest warping during printing due to limited contact with the build plate, while print time and support volume optimisations led to low initial distortions but high residual stress relaxation after support removal. The overheating-optimised orientation was effective in managing thermal stress during the build but produced the highest overall shape deviation. Three-dimensional scanning of the as-printed and post-processed parts, combined with normal surface deviation analysis, confirmed the effectiveness of Atlas 3D predictions and highlighted the importance of evaluating distortions in all spatial directions, especially for complex geometries. Histogram data indicated that most deviations occurred within acceptable ranges, but a small number of critical regions experienced significant warping, reinforcing the need for full-surface inspection. To validate the simulation results experimentally, only the overheating-optimized orientation was manufactured and scanned. This orientation was chosen because it represented the most thermally challenging scenario, providing a robust basis for evaluating Atlas 3D’s prediction accuracy under worst-case distortion conditions.

Beyond distortion, the study assessed the environmental and economic implications of each orientation. The surface area optimisation incurred the highest CO_2_ emissions (900 g/part), energy use, and cost (€832.34/part), primarily due to extensive support structures and longer print times. In contrast, the support volume and print time optimisations achieved the best balance, offering lower emissions, reduced energy demand, and significantly lower production costs (€610–620/part). These findings underscore the close relationship between geometric performance, sustainability, and cost in metal AM. In conclusion, optimal part orientation should not be based solely on mechanical or thermal considerations. A holistic approach, combining distortion minimisation, geometric fidelity, and sustainability metrics, is essential to achieving high-quality and economically viable LPBF components. For the studied motor bracket, the print time and support volume orientations offered the best compromise, highlighting the importance of early-stage simulation and multi-criteria analysis in additive manufacturing process planning.

## Figures and Tables

**Table 1:** Comparison of build parameters for different optimized orientations, obtained from Siemens NX.

Part Orientation Criteria	No. of Layers	Height of Printing (mm)	Support Structures Volume (mm^3^)	Print Time (h)
Surface Area	2787	111.48	368.89	4.68
Support Volume	797	31.88	1366.93	3.57
Print time	725	29	1518.7	3.53
Overheating	2324	92.96	439.01	4.42

**Table 2:** Statistical summary of dimensional deviations measured on the as-built motor bracket.

Parameters	Values
No. of points measured	156,668
Maximum deviation (mm)	0.249
Minimum deviation (mm)	−1.099
Range (mm)	1.349
Mean deviation (mm)	−0.093
Standard deviation (mm)	0.168

**Table 3:** Statistical summary of dimensional deviations measured on the post-processed motor bracket.

Parameters	Values
No. of points measured	305,284
Maximum deviation (mm)	0.294
Minimum deviation (mm)	−0.341
Range (mm)	0.634
Mean deviation (mm)	−0.001
Standard deviation (mm)	0.062

**Table 4:** Comparative summary of the distortions observed across the four orientations.

Optimization Criterion	Build Process Distortion (Z-Direction)	Build Process Distortion (Normal to Surface)	Residual Stress Distortion (Z-Direction)	Residual Stress Distortion (Normal to Surface)	Total Distortion (Z-Direction)	Total Distortion (Normal to Surface)
Surface Area	−0.409 mm to 0.000 mm	−0.392 mm to 0.411 mm	−0.231 mm to 0.198 mm	−0.227 mm to 0.238 mm	−0.227 mm to 0.238 mm	−0.227 mm to 0.238 mm
Support Volume	−0.127 mm to 0.000 mm	−0.143 mm to 0.139 mm	−0.444 mm to 0.237 mm	−0.426 mm to 0.426 mm	−0.402 mm to 0.234 mm	−0.394 mm to 0.379 mm
Print Time	−0.129 mm to 0.002 mm	−0.148 mm to 0.143 mm	−0.472 mm to 0.252 mm	−0.45 mm to 0.455 mm	−0.438 mm to 0.243 mm	−0.426 mm to 0.409 mm
Overheating	−0.324 mm to 0.000 mm	−0.342 mm to 0.331 mm	−0.35 mm to 0.162 mm	−0.381 mm to 0.384 mm	−0.493 mm to 0.229 mm	−0.395 mm to 0.378 mm

**Table 5:** Summary of production cost estimates for each LPBF orientation strategy.

Parameters	Units	LPBF—Surface Area	LPBF—Support Volume	LPBF—Print Time	LPBF—Overheating
Cost	€/Part	832.34	621.78	613.88	785
Cost—per kg	€/kg	23,688.31	17,695.76	17,470.82	22,341.07
Cost—per cm^3^	€/cm^3^	63.25	47.25	46.65	59.65

## Data Availability

The data are included in the article and are available on request from the corresponding author.

## Abbreviations

The following abbreviations are used in this manuscript:

AM	Additive Manufacturing
LPBF	Laser Powder Bed Fusion
CAD	Computer-Aided Design
LCA	Life Cycle Assessment
ISO	International Organization for Standardization
EEA	European Environment Agency
EU-27	European Union (27 member states)
PEF	Primary Energy Factor
CO_2_e	Carbon dioxide equivalent
UNFCCC	United Nations Framework Convention on Climate Change
FEM	Finite Element Modelling
TCN	Thermal Circuit Network
TCE	Thermal Circuit Element
DfAM	Design for Additive Manufacturing
3D	Three-Dimensional
FTR	First-Time-Right
MCDM	Multi-Criteria Decision-Making
ABO	Alternative Build Orientation
OBO	Optimal Build Orientation
E-PBF	Electron Beam Powder Bed Fusion
STL	Stereolithography (file format)
CM	Material Cost
CDP	Data Preparation Cost
MSC	Machine Setup Cost
LMC	Laser Melting Cost
CR	Recoating Cost
CC	Cooldown Cost
CU	Unloading Cost
CS	Separation & Post-processing Cost
CP	Other Process Costs
SR	Support Removal
